# Initial experience with robotic-assisted thoracic surgery for superior mediastinal masses

**DOI:** 10.3389/fsurg.2022.1043525

**Published:** 2023-01-06

**Authors:** Bo Yang, Ruiji Chen, Chengrun Li, Kaijie Fan, Yingxue Lin, Yang Liu

**Affiliations:** ^1^ Department of Thoracic Surgery, First Medical Center, Chinese General Hospital of PLA, Beijing, China; ^2^ Department of Thoracic surgery, Hainan Hospital of Chinese General Hospital of PLA, Sanya, China; ^3^ School of Medicine, Nankai University, Tianjin, China

**Keywords:** robotic-assisted thoracic surgery, mediastinal mass, superior mediastinum, horner's syndrome, mimimally invasive surgery

## Abstract

**Objective:**

Minimally invasive surgery is challenging for masses located in the superior mediastinum, especially for those close to the chest outlet. This study aimed to evaluate the feasibility and safety of robotic-assisted thoracic surgery (RATS) for these masses.

**Methods:**

From June 2015 to January 2020, 35 patients (19 males, 16 females), with a mean age of 41.6 (range, 13–66) years, underwent RATS for the treatment of superior mediastinal masses. Data regarding the operation time, blood loss, pathology, conversion rate, morbidity, mortality, and cost were collected and analyzed.

**Results:**

The mean (±standard deviation) operation time, blood loss, chest tube use duration, and postoperative hospital day were 117 ± 45.2 (range, 60–270) min, 59.7 ± 94.4 (range, 10–500) ml, 4.1 ± 2.1 (range, 1–10) days, and 5.1 ± 2.1 (range, 2–11) days, respectively. The pathological diagnoses included schwannoma (26 cases), ganglioneuroma (4 cases), bronchogenic cysts (3 cases), ectopic nodular goiter (1 case), and cavernous hemangioma (1 case). The mean diameter of the resected tumor was 4.6 ± 2.0 (range, 2.5–10) cm. No conversion or mortality occurred. Postoperative complications included Horner’s syndrome (18 cases: 6 patients with preoperative Horner’s syndrome), weakened muscular power (2 cases), and chylothorax (2 cases). The mean cost was $ 8,868.7 (range, $ 4,951–15,883).

**Conclusions:**

Our experience demonstrated that RATS is safe and feasible for superior mediastinal mass resection. However, the high incidence of postoperative Horner’s syndrome requires further research.

## Introduction

Minimally invasive surgery for mediastinal masses has been widely reportedly comparable to conventional thoracotomy in terms of symptom improvement, recurrence, and survival rate (1, 2). However, surgeons are having difficulty performing video-assisted thoracic surgery (VATS) for masses located at the superior mediastinum, especially for those close to the chest tube outlet due to the narrow complex anatomy, difficulty with hand-eye coordination, and limited movement of VATS instruments.

Robotic surgery provides advantages of instrumentation with 6 degrees of freedom, stable operating arms, and improved visualization with a three-dimensional high-definition camera and has been successfully used to perform mediastinal tumor resection (3, 4). However, studies on RATS for masses in the superior mediastinum are rarely reported.

To our best knowledge, this study included the largest number of masses at this location. Therefore, we aimed to determine the feasibility and safety of robotic-assisted surgery for performing superior mediastinal tumor dissection.

## Materials and methods

### Patients

**Figure 1:**
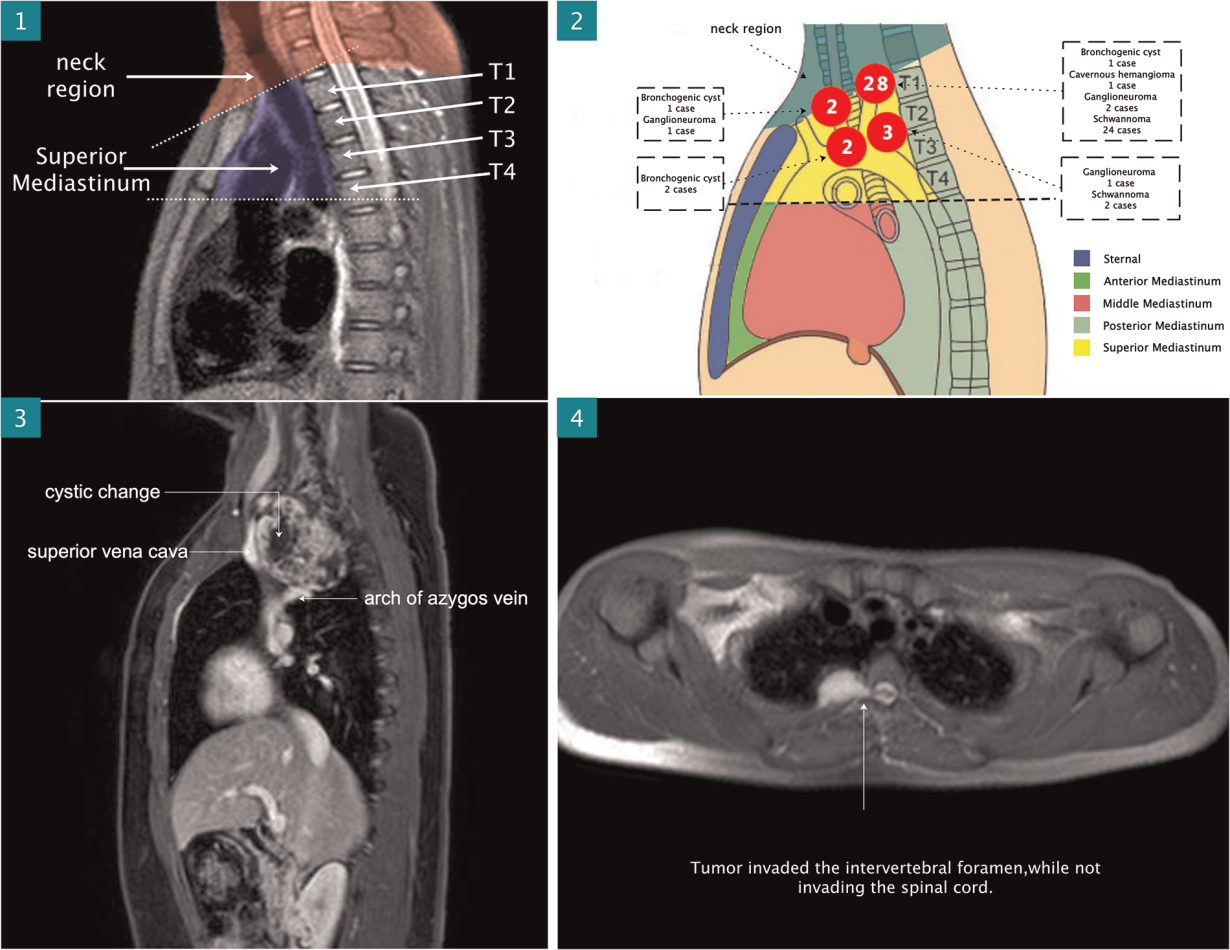
(1) Sagittal MR was used to assess the location of the lesion, which was very helpful in assessing resectability. (2) The schematic diagram shows the location and pathological diagnosis of lesions. (3) Contrast-enhanced thoracic MR was performed preoperatively to exclude neurovascular, chest wall, and vertebral body involvement and intraspinal extension. MR: magnetic resonance.

From June 2015 to January 2020, 35 patients with superior mediastinal masses underwent RATS using the da Vinci S Surgical System (Intuitive Surgical, Inc, Sunnyvale, CA, USA). All of these masses were centrally located above the horizontal plane formed by the sternum angle and the T4–T5 intervertebral discs in a sagittal image. The mass was defined as a cervical-mediastinal mass if its center was further above the horizontal plane formed at the uppermost of the sternal manubrium and T1 vertebra. A contrast-enhanced thoracic magnetic resonance imaging (MRI) sagittal image was routinely obtained preoperatively to confirm (a) the absence of neurovascular, chest wall, or vertebral body involvement and (b) intraspinal extension (Figure 1). Patients aged younger than 16 or over 70 years, those with a mass diameter >10 cm, those with imaging findings that were suspicious of malignancy, and those with MRI signs of invasiveness or intraspinal extension were excluded. A biopsy specimen was not obtained, except for two patients who underwent biopsy in another hospital and were diagnosed with schwannoma. This retrospective review was conducted after obtaining approval from the Institutional Review Board of the Chinese People’s Liberation Army, General Hospital. Informed patient consent was not required because of its retrospective nature.

### Surgical procedure

#### Positioning and anesthesia

The patient was placed in a lateral decubitus position with the lower limbs flexed downward to avoid the hips from interfering with the instrument’s arm movement. Surgery was performed under general anesthesia with one-lung ventilation using a double-lumen endotracheal tube (Covidien IIc, Athlone, Ireland) or bronchial blockers (WELL LEAD MEDICAL CO, LTD, Guangzhou, China).

#### Robot positioning and ports’ layout

**Figure 2:**
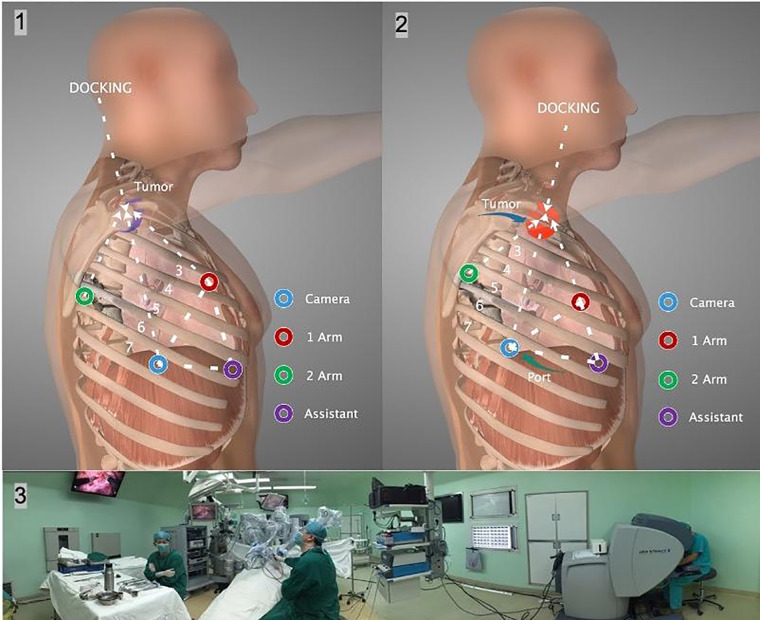
(1), (2) The incision and instrument arm placement depended on the tumor location. Each port was placed at least 5 cm apart from the other ports to prevent instrument arm clashing. The dVS was docked from the head. (3) The panoramic photo shows the intraoperative situation. dVS: Da Vinci system.

The Da Vinci system with three arms was universally used. The robot was docked from the patient’s head. The incision and instrument arm placement depended on the location of masses. The trocar was placed at least 5 cm apart from each other to avoid instrument arm clashing. In 31 patients with masses located posteriorly, the procedure was started with a 12-mm trocar in the 7th intercostal space on the anterior axillary line. A 30° camera was then placed through this port to assess the surgical anatomy and guide the optimal placement of the 8-mm metallic trocar usually at 1 arm in the 6th intercostal space on the posterior axillary line and 2 arms in the 3rd intercostal space on the anterior axillary line. A 12-mm trocar was placed as an assistant port in the 6th intercostal space on the anterior axillary line as needed. In another four patients with masses located anteriorly, the camera port was placed in the 7th intercostal space on the posterior axillary line. The remaining three holes also moved backward as a whole (Figure 2). The general principle is that the center of the mass, operation ports, and camera port together form a diamond shape.

#### Surgeons and surgical technique

**Figure 3:**
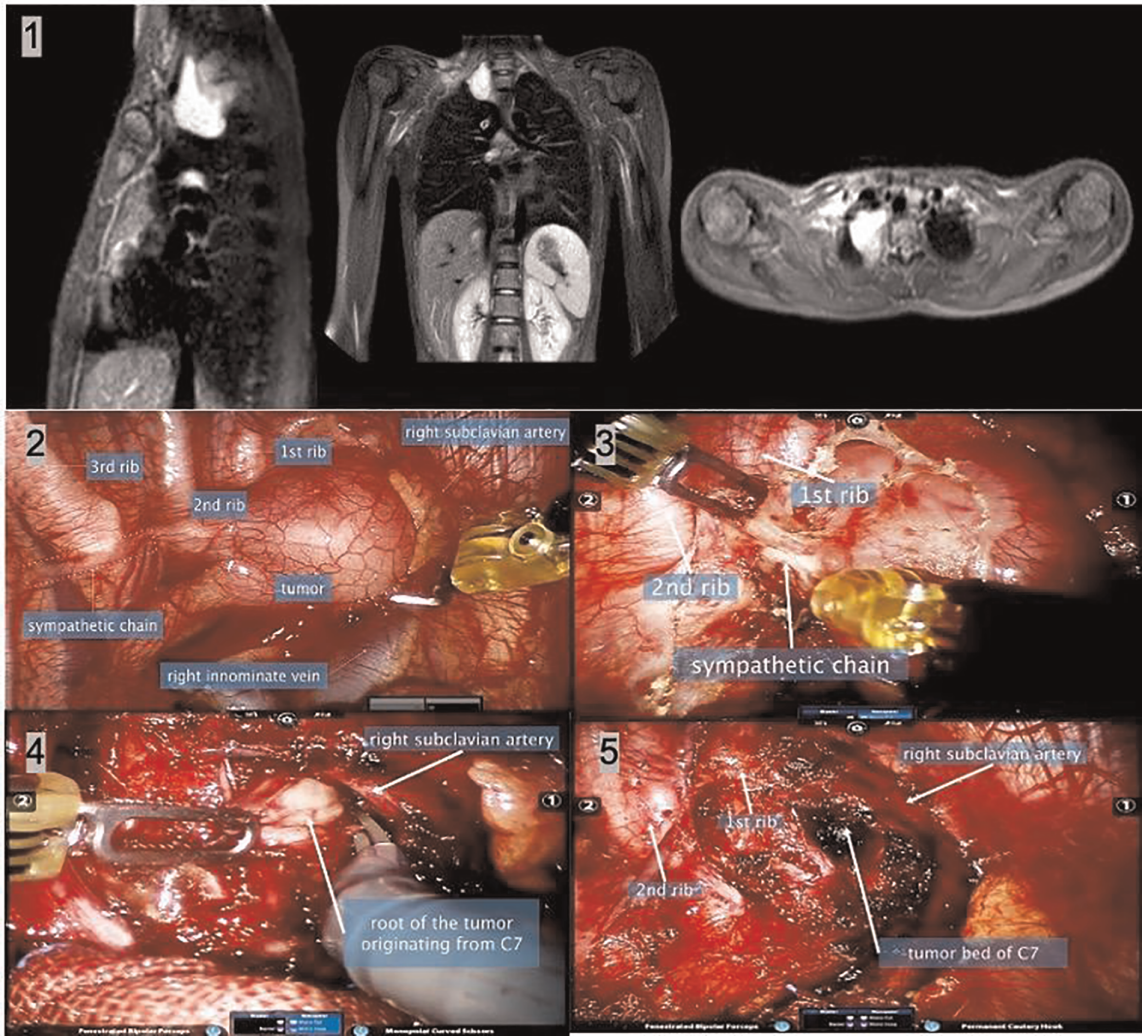
A 13-year-old girl with cervical-mediastinal ganglioneuroma. (1) The thoracic MR shows a lesion located at the right outlet of the chest with a wide base. (2) Intraoperative image shows the lesion and adjacent structures. (3) After separating the mediastinal pleura, the sympathetic nerve chain was found to enter the tumor, which was disconnected, resulting in postoperative Horner’s syndrome. (4) Intraoperative frozen diagnosis was a benign tumor. To fully reveal the root of the tumor, most of the tumors were removed first, and the tumor was found to originate from the C7 nerve root. The root of the tumor was sharply separated with monopolar curved scissors. (5) The image shows the surgical field after complete tumor resection. Postoperatively, the patient developed right upper limb weakness, and symptoms were relieved 2 months postoperatively. MR, magnetic resonance.

Low-flow (8 L/min) carbon dioxide insufflation (8 mmHg) was routinely used. Fenestrated bipolar forceps, a permanent cautery hook, and monopolar curved scissors (Surgical Intuitive, Mountain View, CA, USA) were used to grasp and resect the mass. First, the relationship between the tumor and the sympathetic nerve chain was explored. The tumor was separated along its borders. Extreme care was taken to prevent damage to the subclavian vessels. The use of electrical energy devices near sympathetic nerves was avoided. In the study institution, monopolar instruments were used for tissue dissection, bipolar instruments were used for vascular transection and hemostasis, and scissors were used for sharp dissection (Figure 3).

All specimens were removed with an endoscopic bag after expanding the port in the front (1-arm port), and a 24-Fr chest tube was placed through the camera port for drainage.

All operations were performed by two thoracic surgery specialists (Yang Liu and Bo Yang) who were certified to use the da Vinci Surgical System by the manufacturer.

#### Postoperative management and follow-up

Chest x-ray was performed on postoperative day 1. Our criteria for chest tube discontinuation are (1) drainage of <100 ml per day, (2) no air leakage in the chest cavity, and (3) no active clinical complications. The postoperative follow-up period was 6 months. Symptoms and imaging (CT or MR) are evaluated at 1- and 6-month postoperatively.

#### Data collection and analysis methods

Age, sex, comorbidities, length of surgery, estimated blood loss, length of hospital stay, early and late postoperative complications, conversion to open surgery, pathological diagnosis, and follow-up were reviewed. Operative mortality was defined as death from any cause within 30 days postoperatively or before discharge. The reported mass size was the largest tumor diameter as reported by the pathologist. Data were stored in Excel (Microsoft Corp, Seattle, Wash), and descriptive statistics were shown using the frequency, mean, and standard deviation. Statistical analysis was performed using SPSS 26.0 (IBM, Armonk, NY, USA).

## Results

### Patient characteristics

A total of 35 patients (19 males and 16 females) who underwent robotic-assisted surgery for the treatment of a superior mediastinal mass were included. Their mean age was 41.6 ± 13.5 (range, 13–66) years. The common comorbidities included hypertension (nine patients, 25.7%), diabetes (one patient, 2.9%), and hypothyroidism (one patient, 2.9%). Five patients had a smoking history. Preoperative mass-related symptoms included chest pain (four patients, 11.4%), chest tightness (two patients, 5.7%), Horner’s syndrome (six patients, 17.1%), and brachial plexus compression (four patients, 11.4%, three with upper limb numbness, and one with upper limb weakness). The patient characteristics are shown in Table 1.

**Table 1:** Patient characteristics.

Variables	Value
Sex, male/female	19/16
Age, years	41.6 ± 13.5 (range, 13–66)
Smoking/non-smoking	5/30
Comorbidities	
Hypertension	9 [25.7]
Diabetes	1 [2.9]
Hypothyroidism	1 [2.9]
Symptom	
Horner’s syndrome	6 [17.1]
Chest pain	4 [11.4]
Chest tightness	2 [5.7]
Upper extremity numbness	3 [8.6]
Upper extremity weakness	1 [2.9]

Data are number, number (percentage), or mean ± SD (standard deviation).

### Preoperative imaging

All 35 patients underwent enhanced CT examination preoperatively, and 22 of them additionally underwent enhanced MR examination. A total of 30 tumors were diagnosed as cervical-mediastinal tumors. Pathological diagnosis was issued in 10 CT and 17 MR reports. One patient underwent angiography and embolization due to a large mass and abundant blood supply. The imaging report of four patients (four MR and two CT) showed an enlarged intervertebral foramen. CT reports of seven patients suggested that the masses were closely related to blood vessels; however, none of them were mentioned in MR reports. Preoperative imaging characteristics are shown in Table 2.

**Table 2:** Preoperative imaging characteristics.

Variables	Value
Imaging method	
CT	35
MR	22
CT&MR	22
Mass location	
Superior mediastinum	35
Cervical-mediastinal	30 [85.7]
Pathologic diagnosis and accuracy with postoperative pathology	
CT-Neurogenic tumor	9 [100%]
CT-Bronchial cyst	0
CT-Ectopic thyroid gland	1 [100%]
CT-Cavernous hemangioma	0
MR-Neurogenic tumor	
MR-Bronchial cyst	1 [100%]
MR-Neurogenic tumor	16 [100%]
MR-Cavernous hemangioma	0
Enlarged intervertebral foramen	
CT	4
MR	2
CT and MR	2
Closely related to blood vessels	
CT	7
MR	0
CT and MR	0

Data are numbers or numbers (percentage).

CT, computed tomography; MR, magnetic resonance.

### Surgical procedure

All 35 operations were performed by a robot without conversion to thoracotomy: 20 through the right and 15 through the left thoracic cavity. The mean docking time (the duration from the assistant making the first incision to the surgeon getting to manipulating the robotic arms) was 11.2 ± 3.2 min. The mean operative time (defined as the duration from skin incision until skin closure) was 117 ± 45.2 min, and blood loss was 59.7 ± 94.4 ml. One patient experienced 500 ml of bleeding due to vascular injury to an intercostal artery and received 200 ml blood transfusion during the surgery. Hemostasis was successfully completed using bipolar forceps. The intraoperative characteristics are shown in Table 3.

**Table 3:** Intraoperative characteristics.

Variables	Value
Surgical approach	
Left	15 [42.9]
Right	20 [57.1]
Time	
Docking	11.2 ± 3.2 min
Operation	117 ± 45.2 min
Blood loss	59.7 ± 94.4 ml
Heavy bleeding	1 case [2.9], 500 ml
Blood transfusion	1 case [2.9], 200 ml
Conversion	0

Data are number, number (percentage), or mean ± SD (standard deviation).

### Postoperative outcome, pathology, and follow-up

A total of 18 patients presented with Horner’s syndrome (six with preoperative Horner’s syndrome) and 12 newly occurred (12/29, 41.4%). All three patients with preoperative symptoms of brachial plexus compression were relieved postoperatively. However, two (2/32, 6.25%) new patients developed symptoms of brachial plexus injury (postoperative weakened muscular power). The final diagnosis and postoperative outcomes are shown in Table 2. Complete (R0) resection was accomplished in all patients. No mortality occurred. Chylothorax occurred in two patients, which were both eventually resolved with conservative care.

The mean mass size was 4.6 ± 2.0 (range, 2.5–10) cm. The diagnoses included schwannoma (26 patients), ganglioneuroma (four), bronchogenic cysts (three), ectopic nodular goiter (one), and cavernous hemangioma (one).

The mean chest tube use was 4.1 ± 2.1 (range, 1–10) days, and the postoperative hospital length of stay was 5.1 ± 2.1 (range, 2–11) days. Our hospital uses RMB for settlement, which is equivalent to 8868.8 ± 2207.1 (range, 4,951–15,883) USD based on the 1:6.5 exchange rate.

Follow-up was completed in all patients, and none of them developed local recurrence or distant metastasis. The postoperative characteristics and pathological outcomes are shown in Table 4.

**Table 4:** Postoperative characteristics and pathological outcomes.

Variables	Value
Chest tube use	4.1 ± 2.1 days
Postoperative hospital stay	5.1 ± 2.1 days
Cost	8868.8 ± 2207.1 USD
Postoperative complications	
Horner’s syndrome	12 [41.4]^1^^a^
Upper limb weakness (paralysis)	2 [6.5]^2^^b^
Chylothorax	2 [5.7]
Pathological outcomes	
Mass diameter	4.6 ± 2.0 cm
R0 resection	35 [100]
Schwannoma	26 [74.3]
Ganglioneuroma	4 [11.4]
Bronchogenic cyst	3 [8.6]
Ectopic nodular goiter	1 [2.9]
Cavernous hemangioma	1 [2.9]

Data are number, number (percentage), or mean ± SD (standard deviation).

^a^ 1:6 patients with preoperative Horner’s syndrome were excluded.

^b^ 2:4 patients with preoperative symptoms of brachial plexus compression were excluded.

## Discussion

### Why RATS

In the era of conventional thoracotomy, resection of superior mediastinal tumors often requires supraclavicular incision or sternotomy, which are excessively invasive procedures. With the advent of the minimally invasive era, several approaches have been attempted, some of which still require combined incision (5, 6). In 2010, Tanaka et al. (7) introduced two cases of total thoracoscopic resection of superior mediastinal tumors extending above the thoracic inlet. In the next few years, we used the same procedure in several cases but found that the approach was challenging to perform during the delicate handling required in the thoracic inlet area, the narrowest and farthest, during VATS. Compared to VATS, RATS provides a stable three-dimensional high-resolution view of the surgical field controlled by the surgeon. The EndoWrist operative arm of the dVS can replicate minute human wrist-like movements within the narrow space of the superior mediastinum, the most crucial limitation of the long, rigid instruments of VATS. Moreover, the dVS provides instruments with different functions. Although the instrument arm is also hindered by the intercostal space during surgical procedures such as VATS, the elbow and wrist joints in the thoracic cavity enable a stable and fine operation. However, compared to VATS, the instrument arms require wider trocars, usually 8 mm in diameter. To maintain stability, it also poses greater force on the ribs. These two factors can potentially increase the postoperative incision pain. Besides, the instrument arms occupy more space outside the body and the patient’s arm is stretched to prevent interference, which may also cause postoperative discomfort. Finally, increased cost compared to VATS is another disadvantage. Patients with inferior mediastinal masses were excluded from the present study because these tumors could be conventionally resected *via* VATS. The present study specifically included masses located in the superior mediastinum, which present challenges in VATS. Despite the disadvantages of painful incisions, arm discomfort, and increased costs, we believe that RATS is a safe, smooth, and feasible approach for superior mediastinal masses.

### Key points of RATS

An accurate setup of the dVS was crucial for successful operations (8, 9). The tumor, camera, two robotic arms, and assistant port formed three isosceles triangles at least 5 cm apart from each other to prevent clashing of instrument arms. All operations were performed successfully. A rolled gauze was prepared in case of uncontrolled bleeding. Pressure applied with a rolled gauze helped control the bleeding until an emergency open thoracotomy incision could be performed. For RATS beginners, the operation and docking times progressively decreased. We conducted >100 RATS and passed the learning curve. Therefore, the docking time was constant. In our experience, the fine operation near the intervertebral foramen and the mass isolation with a wide base required the longest time. Decompression of cystic lesions facilitated exposure and shortened the operative time.

### The crucial role of MR in patient selection

The anatomy of the superior mediastinum is complex due to the presence of important neurovascular structures that traverse this area. This has traditionally posed a challenge for surgical access. Due to the difficulty of performing surgery, various surgical methods have been used to achieve adequate exposure (10, 11). To ensure operation safety, preoperative MR examination helps select suitable patients: (1) Assess for vascular/nerve/vertebral involvement (12, 13); (2) The cystic lesions suggested that the tumor could be simplified by decompression; (3) For patients with intraspinal extension, combined thoracoscopic surgery should be considered (14). Enlarged intervertebral foramen on MR should not be considered a contraindication. In this study, four patients with enlarged intervertebral foramen underwent R0 resection. (4) Evaluation of mass blood supply (for masses with abundant blood supply, preoperative embolization can reduce the possibility of intraoperative bleeding). In this study, one patient with an ectopic thyroid gland significantly reduced the blood supply using this method. For patients with intraspinal extension, combined scopic surgery (RATS with tubular retraction system) should be considered. The intraoperative examination of all patients was consistent with the preoperative MR examination, and no vascular/nerve invasion was detected. Based on accurate preoperative imaging evaluation, all surgeries were completed successfully, without conversion to thoracotomy.

### Postoperative outcomes

#### Hospital stay

All patients recovered rapidly and successfully. Compared to other relevant reports, our postoperative hospital stay is significantly longer due to the following reasons: (1) In our hospital, a patient cannot be discharged until the tube was removed. Our criteria for tube removal is drainage of <100 ml/day instead of 200 or 300 ml as required in some hospitals. (2) In this study, the masses were larger, leading to more postoperative exudation. (3) Two patients with postoperative chylothorax were treated and then discharged after conservative management, leading to longer mean postoperative hospital stay (11 and 10 days, respectively).

#### Complications

Nerve injury complications were mainly related to the location of the lesion. Three patients with preoperative brachial plexus compression symptoms were relieved; however, ≥2 patients developed symptoms of brachial plexus injury. All these five masses were cervical-mediastinal tumors. As brachial plexus was invisible in conventional MR and RATS, preoperative FSE-cube sequence MRI examination and intraoperative neural probes may be required to predict and reduce the risk of nerve injury (15).

To prevent Horner’s syndrome, sympathetic nerve chains should be identified and protected first. Although the sympathetic nerve chain was visible in RATS in all patients intraoperatively, the incidence of Horner’s syndrome was higher than in other studies (16, 17). Horner’s syndrome was diagnosed in six patients preoperatively (six with facial hypohidrosis and two with ptosis). The symptoms of these patients were not relieved postoperatively. Twelve patients developed Horner’s syndrome postoperatively (eight with facial and upper limb hypohidrosis, three with upper limb hypohidrosis, and one with ptosis only). Among these, six patients had ptosis. All 18 patients were pathologically diagnosed with neurogenic tumors close to the paravertebral sulcus. Our analyses indicated that, after the exclusion of six patients preoperatively diagnosed with Horner’s syndrome, the mean mass diameter was even smaller in patients with postoperative Horner’s syndrome than in those without it, among the remaining 29 patients. This finding might indicate that the mass location is more significant than the mass diameter in predicting the occurrence of postoperative Horner’s syndrome.

By reviewing the surgical video, we classified 18 patients with sympathetic chain injury into three types based on the relationship between tumor and sympathetic chain. First, no boundary was observed between tumor and sympathetic chain in 11 patients (including all six patients with Horner’s syndrome preoperatively). During these operations, the sympathetic chain could be observed traversing the tumor, suggesting that the tumor may originate from the sympathetic chain. Such patients may be unable to avoid Horner’s syndrome. Second, the sympathetic chain was adjacent to the tumor in five patients. Although the sympathetic chain was preserved intraoperatively, Horner’s syndrome still occurred postoperatively. This may be related to the use of intraoperative electrical energy devices. Third, the tumor was far from the sympathetic chain in two patients, which may be related to the stimulation of the sympathetic chain intraoperatively. Indeed, the sympathetic chain is easily damaged and stimulation of the thoracic drainage tube can cause Horner’s syndrome.

The increased incidence of Horner’s syndrome may be caused by surgical approach changes. In open surgery, the tumor is first isolated from the surrounding tissue, and the deepest root is finally treated. Unless the tumor originates from the sympathetic chain, most can be separated bluntly, and the risk of nerve injury is low. Conversely, in RATS surgery, the tumor base is first exposed and can only be pulled near aside by breaking off the tumor base. When the tumor base is closely related to the sympathetic chain, the dissociation process may increase the risk of sympathetic nerve injury. Therefore, careful identification and protection of the sympathetic nerve chain intraoperatively and avoidance of clamping and using electrical energy devices around the sympathetic nerve chain are keys to preventing the occurrence of Horner’s syndrome.

### Cost

The mean cost was $ 8,868.8 ± 2,207.1 (range, $ 4,951–15,883), which was comparable with another study (4). The cost-effectiveness of this technique was not within the scope of this study, and thus, further studies regarding this issue are needed.

### Lack of research

The was a single-center retrospective study and was not feasible to conduct a randomized controlled study due to the low incidence of the disease. However, to our knowledge, this is the largest series report on RATS for superior mediastinal masses.

## Conclusions

In preoperative MR, appropriate patients can be selected for safe robotic surgery. More attention should be paid to protecting nerves intraoperatively to prevent complications.

Due to surgical approach changes, robotic surgery may increase the risk of sympathetic nerve chain injury, leading to an increased incidence of Horner’s syndrome, which should be closely considered intraoperatively.

## Data Availability

The original contributions presented in the study are included in the article/Supplementary Material, further inquiries can be directed to the corresponding author/s.
